# Dynamical X-ray diffraction imaging of voids in dislocation-free high-purity germanium single crystals

**DOI:** 10.1107/S1600576720005993

**Published:** 2020-06-12

**Authors:** Kevin-P. Gradwohl, Andreas N. Danilewsky, Melissa Roder, Martin Schmidbauer, József Janicskó-Csáthy, Alexander Gybin, Nikolay Abrosimov, R. Radhakrishnan Sumathi

**Affiliations:** a Leibniz-Institut für Kristallzüchtung, Max-Born-Strasse 2, 12489 Berlin, Germany; b Kristallographie, ALU Freiburg, Hermann-Herder-Strasse 5, 79104 Freiburg, Germany

**Keywords:** X-ray topography, dynamical theory, high-purity germanium, vacancies, voids, dislocation density, diffraction contrast

## Abstract

White-beam X-ray topography has been performed to provide direct evidence of micro-voids in dislocation-free high-purity germanium single crystals. The voids are visible because of a dynamical diffraction contrast. It is shown that voids occur only in dislocation-free parts of the crystal and do not show up in regions with homogeneous and moderate dislocation density. It is further suggested that the voids originate from clustering of vacancies during the growth process. A general method is proposed to verify the presence of voids for any crystalline material of high structural perfection.

## Abstract 2

White beam X-ray topography has been performed to provide direct evidence of micro-voids in dislocation-free high-purity germanium single crystals. A general method is proposed to verify the presence of voids for any crystalline material of high structural perfection.

## 1. Introduction   

Recently, there has been an enormous demand for highly sensitive radiation detectors made of germanium single crystals. These detectors are utilized in the search for a very rare lepton-number-violating nuclear event, called the neutrinoless double-beta decay (Abgrall *et al.*, 2017 ▸). The germanium required for these radiation detectors should be of high crystalline perfection. More precisely, a low net charge carrier density [high-purity germanium (HPGe)] and a homogeneous distribution of a low number of structural defects such as dislocations and micro-voids (voids) are required (Hansen & Haller, 1972 ▸). Voids, especially, are very difficult to detect since they typically occur in low densities and are small in size.

We perform white-beam X-ray imaging and use dynamical diffraction imaging to show direct evidence for voids in dislocation-free HPGe. Both Ge and Si single crystals have been grown dislocation free for decades (Dash, 1959 ▸; Tweet, 1958 ▸). Comparable investigations are reported for nearly perfect silicon crystals by Deslattes *et al.* (1999 ▸) and Tuomi *et al.* (2001 ▸). However, HPGe has specific material properties allowing one to conclude without any doubt that the localized tensile strain fields detected by X-ray topography are in fact vacancy clusters in the form of voids. In Si, vacancies and self-interstitials can occur in equal concentrations at thermodynamic equilibrium at the melting point, whereas in Ge, vacancies are always the dominant species. It has been shown by first-principles calculation (Śpiewak *et al.*, 2007 ▸), as well as experimentally (Vanhellemont *et al.*, 2007 ▸), that the vacancy concentration at the melting point of Ge is of the order of 10^14^–10^15^ cm^−3^ depending on the electrical charge of the vacancy. This is several orders of magnitude larger than the concentrations of self-interstitials (10^9^ cm^−3^) and impurities (<10^12^ cm^−3^). So, in dislocation-free Si, the annihilation of intrinsic point defects (vacancies and self-interstitials) might be sufficient to reduce the vacancies, while in large dislocation-free Ge crystals, vacancies cannot be completely annihilated, ultimately cluster after the crystal has been cooled from the melting point and cause a range of vacancy-related crystal defects (Haller *et al.*, 1981 ▸). Since in Ge the effective vacancy sink is the absorption of vacancies along the dislocation line (decorating the dislocation), no voids are expected in parts of the crystal with moderate dislocation density. Therefore, we show here that the voids are only observed in dislocation-free parts of the crystal, whereas no voids are observed in parts of the crystal with a homogeneous dislocation density of ∼2600 cm^−2^.

## 2. Experimental   

Single-crystalline HPGe (net charge carrier density <10^12^ cm^−3^) was grown by the Czochralski method using zone refined Ge bars with high purity, as described in great detail elsewhere (Abrosimov *et al.*, 2020 ▸). The investigated crystals were grown in the [001] direction under different gas atmospheres. One crystal was grown under constant Ar flow, while the other was grown under a constant flow of ultra-pure H_2_ purified in a Pd cell. For radiation-detector applications, a low and homogeneous dislocation density between 10^2^ and 10^4^ cm^−2^ is required (Wang *et al.*, 2014 ▸). Therefore, a Dash neck procedure was used to get rid of the initial dislocations. The temperature gradients within the growth setup and the growth parameters are controlled to ensure low dislocation densities. This has been assured by various crystal growth experiments and a detailed simulation of the temperature and stress field during growth, which has been used to optimize the crystal growth apparatus (Miller *et al.*, 2020 ▸). Along the growth direction of these crystals, wafers with (001) surface orientation and with thicknesses ranging from 350 to 700 µm were cut. They were chemo-mechanically polished on both sides for X-ray topography investigations.

**Figure 1:**
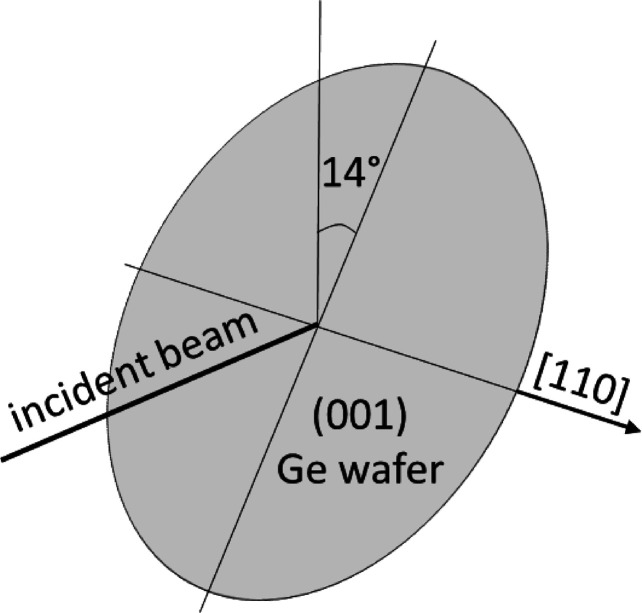
The measurement geometry of the (001) Ge wafers, which were tilted around the [110] direction by 14° to obtain the 400 and 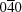 stereo pair as well as the 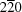 topograph on one film.

White-beam X-ray imaging was conducted at the topography station at the imaging cluster of the Karlsruhe Research Accelerator (KARA) synchrotron – a 2.5 GeV electron storage ring situated at the Karlsruhe Institute of Technology, Germany. The detailed experimental setup of the beamline is described elsewhere (Rack *et al.*, 2009 ▸). The wafers were measured in transmission geometry with typical exposures of several minutes. A two-dimensional detector with a pixel size of 2.5 µm and a Slavich high-resolution photographic film (VRP-M) with a size of ∼100 × 125 mm were used to record the topographs. The distance between the sample and the high-resolution photographic film was 95 mm. The samples were tilted by an angle of 14° around the [110] direction (Fig. 1 ▸) to obtain the 400 and 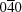 stereo pair, as well as the 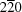 topograph on one film. For the investigation of the 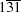 topograph the sample was tilted around the [110] axis by an angle of 28°.

## 3. Results and discussion   

### 3.1. Voids in crystals grown in Ar   

**Figure 2:**
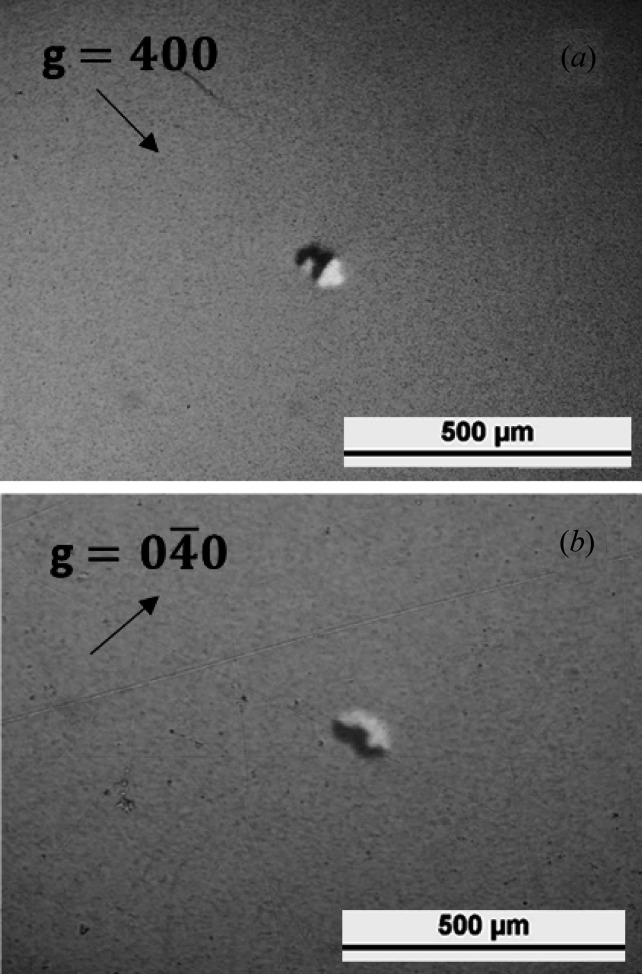
A stereo pair of synchrotron transmission topographs of voids in dislocation-free HPGe recorded at the topography station at the imaging cluster of the KARA synchrotron. The voids are situated close to the exit surface of the beam and are only visible because of a dynamical diffraction phenomenon.

A 400 and 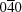 stereo pair of Ge grown in Ar atmosphere measured in transmission from the same X-ray film is presented in Fig. 2 ▸. The two round slightly overlapping features with a diameter of ∼60 µm are remarkable. They show a black–white contrast following the diffraction vector, where black indicates high intensity and white indicates low intensity. The small black dots in both topographs are defects in the X-ray film itself and the small line at the top of the 400 topograph [Fig. 2 ▸(*b*)] is an artifact related to the light path of the optical microscope used to analyze the films. The total area investigated by X-ray topography was of the order of cm^2^ and is dislocation free.

The Miller indices of the topographs were determined by simulating Laue diffraction patterns using *LauePt* (Huang, 2010 ▸) and matching them to the films. Afterwards, the fundamental excitation wavelength of the topographs was determined to be λ = 0.52 Å (*E* = 23.8 keV) and λ = 0.45 Å (*E* = 27.6 keV) for the 400 and 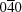 topographs, respectively. This slight asymmetry is caused by the offcut angle to the [001] direction of ∼1°, which might originate from the thinning of wafers by a grinding process. We propose that the black–white image in the topographs is related to localized tensile strain fields close to the exit surface of the crystal. In the crystals grown in this study, the black–white contrast can only be explained by vacancy clusters in the form of voids.

### 3.2. Imaging principle of voids   

We follow the reasoning of Authier & Malgrange (1998 ▸) and Deslattes *et al.* (1999 ▸) to give a qualitative explanation for the observation of the black–white contrast. The two-beam case of dynamical diffraction theory is applied to describe the experimental observations, which is justified by the high crystalline perfection of the crystal, namely being single crystalline, dislocation free and of high purity. In this picture, the beams inside the crystal interfere with each other, exchanging energy, leading to two dispersion branches separated by the forbidden Bragg gap (Tanner, 1976 ▸; Authier, 2010 ▸). The wavefields related to branch 1 of the dispersion surface undergo anomalous absorption, while the wavefields of branch 2 are strongly absorbed. The product of the absorption coefficient μ_0_ and the wafer thickness *t* (350 µm) is 4.6 for the 400 topograph and 3.4 for the 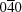 topograph (Macgillavry *et al.*, 1962 ▸), which is sufficiently high to omit the wavefields of branch 2 in our investigations. Regarding the anomalous transmission of branch 1, the product of the anomalous absorption coefficient μ_*i*_ and the thickness of the wafer is 0.17 and 0.13 [estimated from Persson & Efimov (1970 ▸)] for 400 and 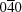, respectively. Therefore, in the following argument only the wavefield related to branch 1 is considered.

**Figure 3:**
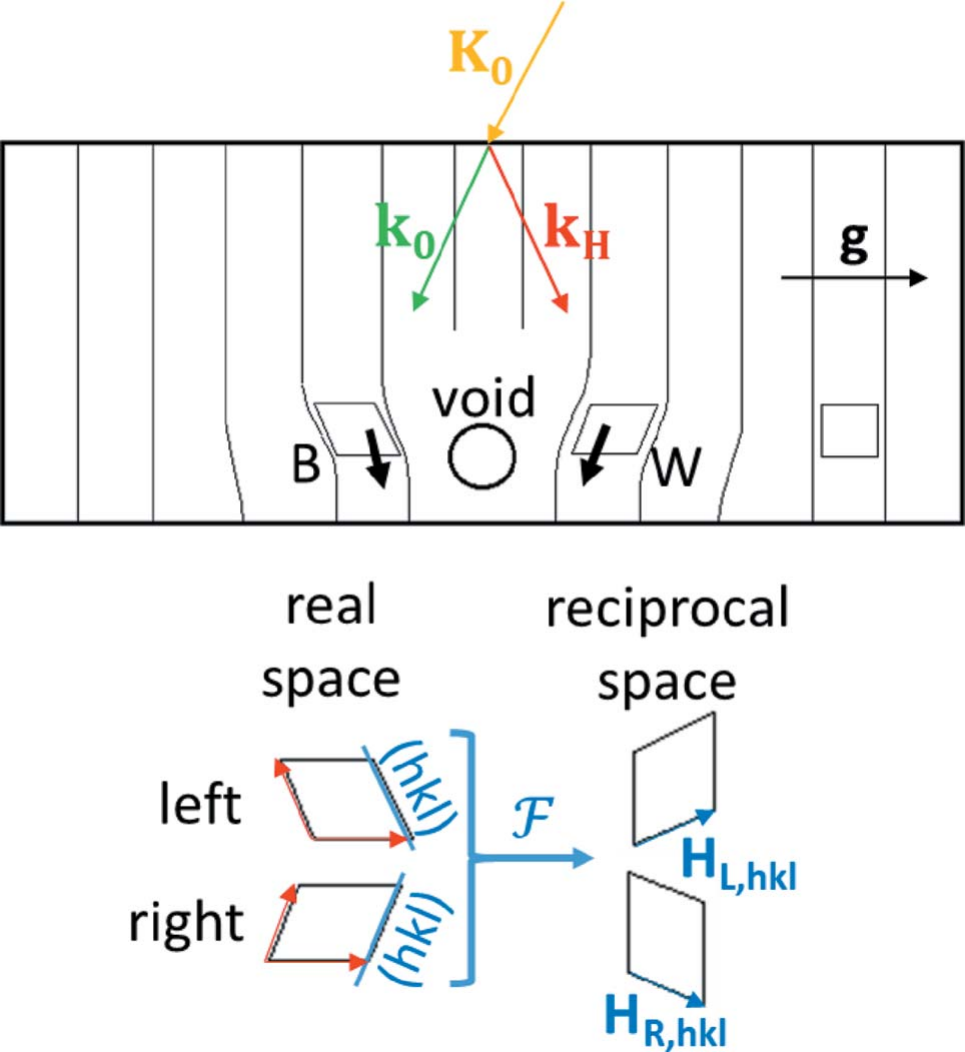
Ge crystal with distorted lattice, owing to a void close to its exit surface. The diffraction vector is indicated by **g**, the wavevector **K**_0_ represents the incident beam, and *k*_0_ and *k*_h_ represents the transmitted and the diffracted beam, respectively. The short black arrows labeled B (black image) and W (white image) indicate the direction of energy flow of the wavefields left and right of the void. A schematic transformation of the distorted lattice close to the void into reciprocal space is shown, indicating the distorted reciprocal-space vectors left and right of the void **H**_L, *hkl*_ and **H**_R, *hkl*_.

**Figure 4:**
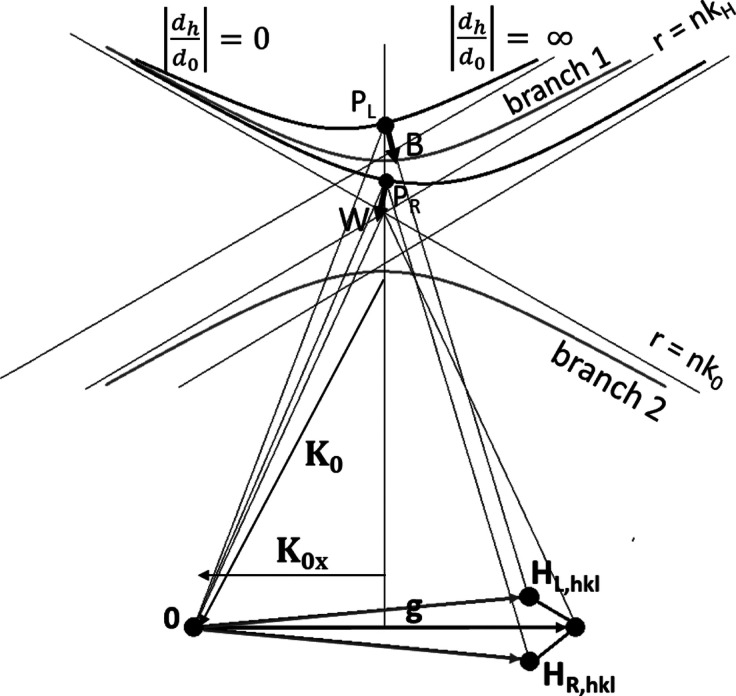
The relationship between the changes of the dispersion surfaces with a change of reciprocal lattice vectors associated with a distorted lattice. The shift of the tie points relative to the vertex of the dispersion surface explains the change in intensity left and right of the void.

The crystal lattice around the void is distorted and bends inwards in close proximity to the void as a result of its tensile stress field. The deformations cause a strongly increased absorption, since the effective absorption coefficient depends on the deviation from the precise Bragg condition. However, if the void is situated close to the exit surface of the crystal (Fig. 3 ▸), the increased absorption caused by the distorted lattice is negligible. The deformations are so small that locally the crystal is still perfect enough to apply the dynamical diffraction theory (Penning & Polder, 1961 ▸). The local tensile strain fields tilt the lattice planes (*h* *k* *l*) towards the void, which leads to a local variation in the reciprocal lattice vectors δ**H**_L, *hkl*_ and δ**H**_R, *hkl*_ (L = left, R = right), as can be seen in Fig. 4 ▸. To a first approximation, the structure factor does not change and consequently the Lorentz point of the deformed lattice must lie on the sphere with origin 0 and radius *r* = *n* *k*_0_. Subsequently, the whole dispersion surface shifts up left of the void and shifts down right of it, as indicated in Fig. 4 ▸, owing to the local deformations if the void is situated close to the exit surface. The bold arrows at the tie points of the deformed lattices are the Poynting vectors of the wavefield, **P**_R_ and **P**_L_. The associated wavefields must fulfill the continuity of the tangential components of the wavevectors at the crystal surface.

The tie point **P**_L_ is shifted to the right relative to the vertex of the dispersion surface, while **P**_R_ is shifted to the left. The ratio of the diffracted amplitude relative to the transmitted one depends on the position of the tie point relative to the vertex of the dispersion surface, indicated by 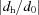, where *d*_h_ is the electric displacement field of the diffracted beam and *d*_0_ is the electric displacement field of the transmitted beam. Within the hyperbolic approximation of the dispersion surface close to the Bragg gap, the amplitude ratio 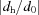 vanishes left of the void, while it tends towards infinity right of it. Therefore, an increased intensity of the diffracted beam is observed left of the void (B for black image), while right of it the intensity is decreased (W for white image). As a consequence, a black–white contrast following the diffraction vector **g** can be observed.

Therefore, the black–white contrast can be described as a modulation of diffraction intensity caused by a change in the tie point position, owing to the inward bending of lattice planes next to local tensile strain fields close to the exit surface of the crystal.

### 3.3. Voids in crystals grown in H_2_   

White-beam X-ray topographs of two (001) wafers, cut from the top and the tail part of an HPGe crystal grown under constant H_2_ flow were recorded.

**Figure 5:**
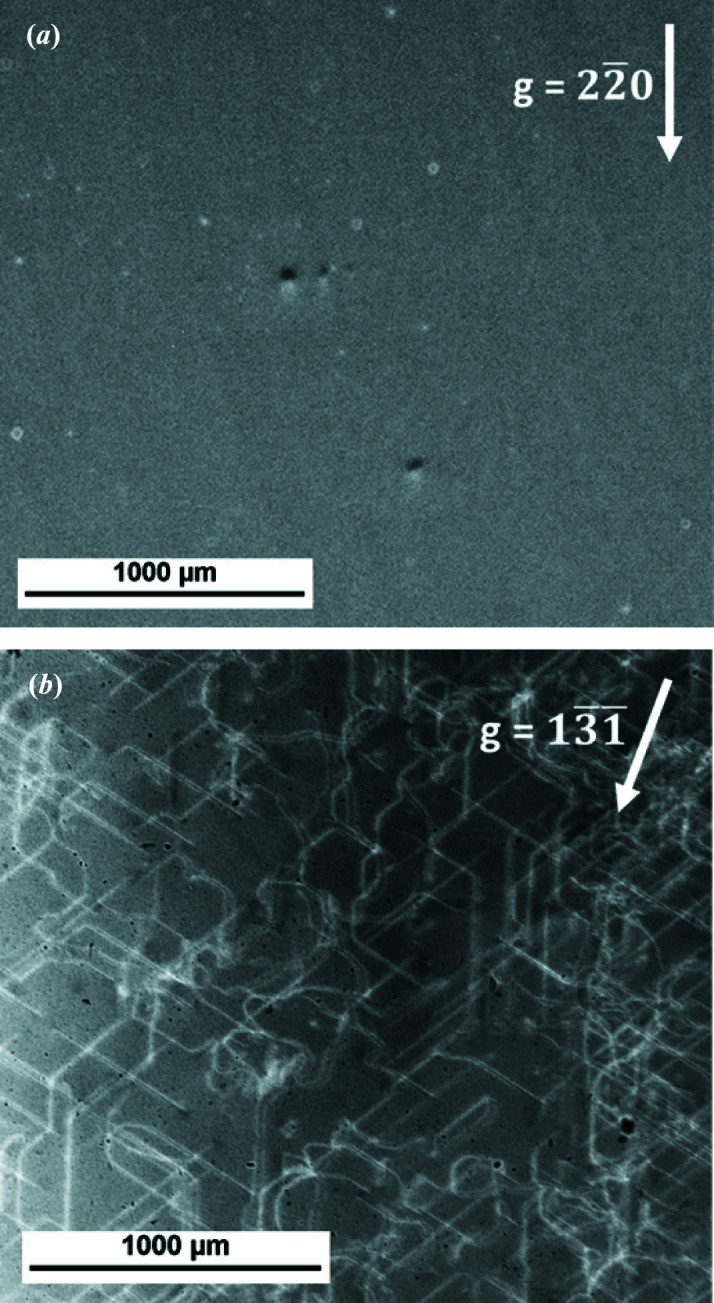
(*a*) The 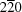 topograph of the top end of the crystal grown in H_2_ shows the crystal to be free of dislocations and contains a few of the striking features with a black–white contrast indicating the presence of voids. (*b*) The 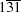 topograph of the same crystal (tail part), revealing a dislocation density of 2600 cm^−2^ and showing no sign of voids.

The top part of the crystal was measured with a low-resolution film to find the exact position of the Bragg reflections, and then 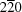 was chosen for a larger-scale (whole wafer) investigation with a two-dimensional detector [Fig. 5 ▸(*a*)], since this reflection can be reached by simply tilting the sample around the horizontal [110] axis. No dislocations can be seen in the presented topograph, but the dislocation density was found to be 1 cm^−2^ by evaluating the X-ray topographs on the whole wafer scale. The long and thin Dash neck eliminated most of the dislocations in the top part of the crystal. The small white spots are detector artifacts. There were also no noticeable dislocations in the topographs recorded on the low-resolution film. In this dislocation-free part of the crystal, a number of spherical black–white features can be observed. The black–white contrast with a diameter of 50 µm follows the diffraction vector again and indicates the presence of voids in the crystal. Throughout the entire wafer map, several more voids could be detected. The size of the voids could in principle be determined by simulating the dynamical diffraction pattern for varying void sizes for the given experimental setup, which should be consistent with the atomistic simulations of the vacancy-clustering dynamics of the cooling process of the crystal.

In contrast, the wafer from the crystal tail has a moderate homogeneous dislocation density of ∼2600 cm^−2^, forming characteristic pseudo-hexagonal loops with dislocation lines strictly oriented along the 〈110〉 directions of the crystal, as can be seen in the 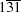 topograph of Fig. 5 ▸(*b*). The dislocation density was estimated by measuring the total length of dislocation lines in the topographs and dividing it by the measurement volume, based on the sample thickness and measured area. The lines in the topograph represent single dislocation lines, and the average length of undisturbed straight dislocation line segments is of the order of several hundred micrometres. In the right section of Fig. 5 ▸(*b*), dislocation lines start to interact, forming dislocation bundles. However, the total length of the dislocation lines could be determined with sufficient accuracy. The small black dots are again defects in the film. As expected and argued before, no voids can be found in any regions of the crystal with significant dislocation density. In fact, in all our investigations, the black–white contrast indicating voids could be only found in dislocation-free parts of the crystals, indicating vacancy-related defects. Precipitates or inclusions usually induce a compressive strain field, which forms an opposite white–black contrast following the diffraction vector. This demonstrates that the observed black–white contrast is directly related to point defect agglomeration occurring in dislocation-free parts of the crystal only, depicting voids and not other defects such as precipitates.

## 4. Conclusions   

It was possible to show the presence of voids in dislocation-free parts of Ge crystals grown under different conditions by white-beam X-ray topography. These voids are not easily detectable by other methods since they are too small for optical microscopy, but they appear in such low densities (estimated density below 10^5^ cm^−3^) that they are also not detectable by electron microscopy. X-ray topography in this regard is the perfect characterization technique since it combines a large field of view, of the order of mm^2^ to cm^2^, with a high sensitivity to the strain field of the voids, which is much larger than the defect itself. This is due to the sensitivity of the dynamical diffraction effect to the bending of the lattice planes and ultimately to the strain field of the voids, which is significantly more extended than the void itself.

Furthermore, it was possible to confirm the observations of Deslattes *et al.* (1999 ▸) and Tuomi *et al.* (2001 ▸) on nearly perfect Si crystals. They were not able to show that the observed dynamical diffraction patterns are in fact caused by voids, which we can support with this work. We should also state that compressive strain fields from, for instance, precipitates or inclusions can cause an opposite white–black contrast following the diffraction vector, as has been shown for a rare earth vanadate (Tanner, 1976 ▸).

Finally, we propose a general technique based on white beam X-ray topography which can be used to detect voids in any crystalline material with high crystalline perfection. The theoretical description of the black–white contrast based on dynamical diffraction theory does not assume any material-specific properties and is therefore universal for any crystalline material, which was shown experimentally for Si crystals and here for HPGe crystal grown under different atmospheres.
